# Neonatal intestinal obstruction due to double jejunal web causing Windsock deformity

**DOI:** 10.4103/0971-9261.71751

**Published:** 2010

**Authors:** Aejaz A. Baba, Altaf H. Shera, Afak Y. Sherwani, Iftikar H. Bakshi

**Affiliations:** Department of Pediatric Surgery, SKIMS, Srinagar, J&K, India

**Keywords:** Intestinal obstruction, jejunal web, Windsock deformity

## Abstract

Windsock deformity (WD) is a rare anomaly. A case of double jejunal web with WD causing neonatal intestinal obstruction is being reported.

## INTRODUCTION

Jejuno-ileal atresias have been categorized into type I (mucosal web), type II (atretic fibrous cord), type IIIa (V-shaped mesenteric defect), type IIIb (‘apple peel atresia’), and type IV (multiple atresias).[1] Windsock deformity (WD) is a rare anomaly. The second part of the duodenum is the most common site of WD. We report a unique case of double jejunal web with WD in a neonate.

## CASE REPORT

A three-day-old full-term male neonate was admitted with a history of abdominal distension and bilious vomiting since birth. The baby had not passed meconium since birth. Epigastric distension was noted on abdominal examination. The abdominal radiograph revealed a few gaseous shadows in the proximal gut and completely gasless lower abdomen. An ultrasonography and echocardiography ruled out any associated genito-urinary and cardiac anomalies.

**Figure 1:**
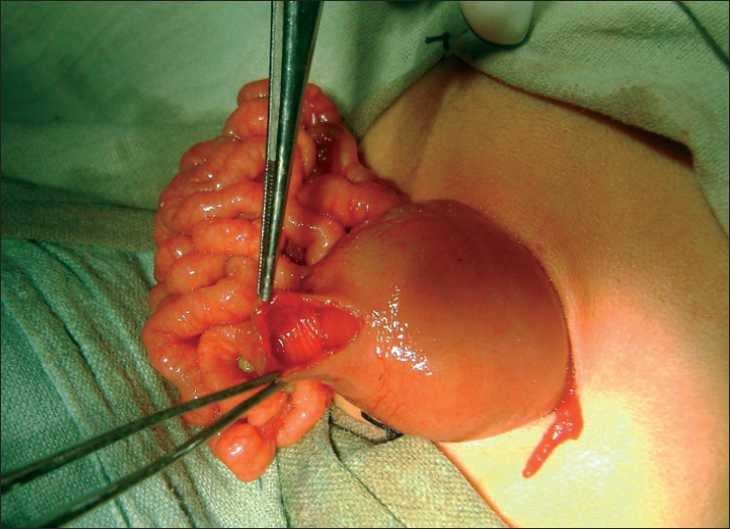
Proximal web seen after enterotomy

**Figure 2:**
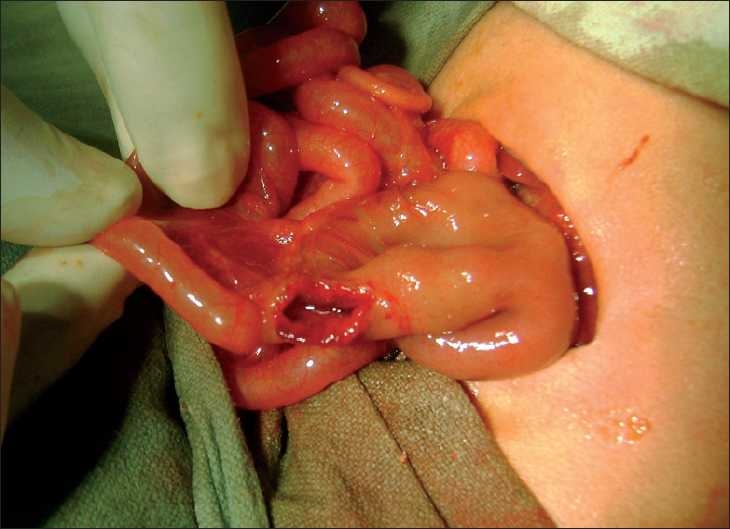
Another web 2 cm distal to the proximal web visible after saline injection

At laparotomy, dilatation of the proximal 5 cm of jejunum, duodenum, and stomach were seen. The small gut loops distal to the obstruction were completely collapsed. A longitudinal enterotomy was performed just distal to the site of obstruction along the antimesenteric border. A windsock web causing total obstruction of the jejunum was noticed proximal to the enterotomy site [Figure 1]. The web was excised circumferentially and the proximal bowel deflated. A second simple web completely obstructing the jejunal lumen [Figure 2] was noticed 2 cm distal to the enterotomy site after the saline test to check the patency of distal bowel was performed. This web was also excised from the same enterotomy site. The enterotomy site was closed. Post-operatively the patient made a delayed recovery with bowel functioning on the 14th postoperative day. The patient was thriving at 6 months follow-up.

## DISCUSSION

Windsock deformity of the jejunum due to congenital web is a very rare anomaly with scant literature. Furthermore, to the best of our knowledge, the presence of two webs in the jejunum has not been reported till date in the English literature and hence the importance of this case.

Andrews and Stem (1981) reported a case of jejunal web in a 48-h old Arab female neonate.[2] The newborn had presented with small bowel obstruction, upper abdominal distention, and bile-stained vomitus. A jejunal web was removed during surgery and jejunoplasty performed. De Backer *et al*. treated a one-year-old boy with high jejunal membranous stenosis successfully by antimesenteric longitudinal enterotomy over the diaphragm, excision of the latter, and transverse closure of the bowel.[3] Kothari *et al*. reported a case of jejunal web with a central perforation situated 8 cm from the duodeno-jejunal junction in a four-year-old emaciated male child with history of intermittent episodes of bilious vomiting, abdominal pain, and failure to thrive. Enterotomy with excision of web was done and the child had an uneventful post-operative recovery.[4] Seltz reported a case of jejunal web with a pinhole in a 13-month-old boy with a history of failure to thrive and recurrent episodes of non-bilious emesis beginning at six months of age. Surgical excision of the web was done without any complications.[5] Our case can be included into type IV jejunal atresia because of presence of multiple atresias.

The treatment for jejunal web remains surgical excision even though variable approaches may be used. Endoscopic laser therapy has been successfully tried for duodenal web. Simultaneous laparotomy with endoscopy has also proved useful.[3] Our patient underwent enterotomy with excision of the webs.
